# A model to explain smokeless tobacco consumption in adults: A grounded theory study

**DOI:** 10.1016/j.heliyon.2023.e20734

**Published:** 2023-10-13

**Authors:** Esmaeil Fattahi, Mahnaz Solhi, Seyed Saeed Hashemi Nazari, Hadis Barati, Fardin Mehrabian, Zahra Sadat Manzari, Iraj Zareban, Abolhasan Afkar

**Affiliations:** a Department of Health Education and Promotion, School of Health, Guilan University of Medical Sciences, Rasht, Iran; b Department of Education and Health Promotion, School of Health, Iran University of Medical Sciences, Tehran, Iran; c Department of Epidemiology, School of Public Health and Safety, Shahid Beheshti University of Medical Sciences, Tehran, Iran; d Department of Health Education and Promotion, Research Center of Health and Environment, School of Health, Guilan University of Medical Sciences, Rasht, Iran; e Department of Medical-Surgical Nursing, School of Nursing and Midwifery, Mashhad University of Medical Sciences, Mashhad, Iran; f Health Promotion Research Centre, Zahedan University of Medical Sciences, Zahedan, Iran; g Research Center of Health and Environment, School of Health, Guilan University of Medical Sciences, Rasht, Iran

**Keywords:** Smokeless tobacco, Adults, Qualitative research, Grounded theory, Model, Concepts

## Abstract

**Introduction:**

Smokeless tobacco use remains a significant public health concern, necessitating the acquisition of comprehensive and extensive data to effectively address and control its consumption. Understanding the underlying patterns of consumption is crucial for this purpose.

**Objective:**

This study aimed to develop a model that explains smokeless tobacco consumption among adults in the southeastern province of Iran, specifically in the city of Chabahar.

**Methods:**

A qualitative study was conducted using a grounded theory approach with inductive processes. The participants consisted of 30 adults aged 18–64 years from Chabahar City in southeastern Iran. Purposive sampling was used to select participants, and data collection continued until theoretical saturation was achieved. Data analysis followed Strauss and Corbin's perspective, involving four stages: Analyzing Data for Concepts, Analyzing Data for Context, Bringing Process into the Analysis, and Integrating Categories.

**Results:**

The analysis of data yielded three main categories: “starting to use,” “continued use,” and “cessation attempts,” each with their respective subcategories. Additionally, two main categories related to the consequences of smokeless tobacco consumption were identified: “addiction and efforts to overcome addiction,” also with their respective subcategories.

**Conclusion:**

The Dependency Cycle Model in Consumption provides a comprehensive understanding of the contextual factors, processes, and consequences associated with smokeless tobacco consumption. This model serves as a valuable tool for researchers aiming to develop effective interventions in the field of smokeless tobacco consumption.

## 1 Introduction

Smokeless tobacco refers to products that are consumed without burning or smoking. It includes various forms such as chewing tobacco, snuff, snus, and dissolvable tobacco. Users typically place the product in their mouths, allowing nicotine to be absorbed through the oral mucosa. The consumption of smokeless tobacco poses a significant public health problem and challenge [1]. And it has numerous harmful side effects. These include oral health issues, oral lesions, an increased risk of cancer, nicotine addiction, cardiovascular risks, reproductive health problems, oral discomfort, bad breath, and social stigma. In communities where this health problem persists, researchers are constantly seeking ways to reduce the consumption of these substances among consumers [2,3]. The lack of in-depth knowledge regarding the nature of these substances and the absence of information about the potential reasons for their use [[4], [5], [6], [7], [8], [9]] have made it somewhat challenging to prevent and implement effective interventions to reduce their consumption. Some studies have highlighted the role of social, cultural, and environmental factors [[10], [11], [12]]. According to numerous studies, the use of these substances has increased in certain communities and age groups, spanning from childhood to old age, and affecting both men and women. Although the harms associated with using these substances are evident to most consumers [6,[13], [14], [15]], it is important to investigate the factors that contribute to their use or non-use [5,6,[16], [17], [18]].

Grounded theory methodology offers a reliable set of procedures for constructing theories based on data. These procedures allow researchers to explore various aspects of topics and related behaviors, thus developing comprehensive explanations. They can be applied to gain fresh insights into long-standing issues, as well as to investigate emerging areas that require further examination. The procedures help uncover underlying beliefs and meanings that drive actions, analyze both rational and nonrational aspects of behavior, and illustrate how logic and emotion intertwine to shape individuals' responses to events or problem-solving through action and interaction. Moreover, grounded theory methods facilitate the development of both specific and more general theories [[19], [20], [21]].

Given the complexity and multitude of factors associated with smokeless tobacco consumption across all age groups, our objective is to develop a model that effectively explains the process of this consumption. Employing a qualitative method will enable us to achieve this goal. One widely used qualitative approach among researchers is the grounded theory approach introduced by Strauss and Corbin in 2008 [19,20].

Grounded Theory is an inductive research approach that involves generating hypotheses and theories based on collected data. It is a systematic method of gathering and analyzing data to develop theories about patterns of human behavior within social contexts [22]. This approach provides a systematic means to uncover profound and significant information that quantitative methods often cannot capture. It also allows for the emergence of new concepts, ultimately leading to the development of an integrated theory [20,23].

The qualitative approach employed in this study is valuable in addressing the complexities and research questions of the subject matter. The objective of this research was to develop a model that explains smokeless tobacco consumption among adults in the southeastern province of Iran, specifically in the city of Chabahar.

## 2 Methods

### 2.1 Study setting

In this study, we utilize the grounded theory approach introduced by Strauss and Corbin in 2008 [19]. This approach places emphasis on systematic analysis to uncover the underlying processes of social interactions. Our main objective is to present in-depth qualitative findings and outline the systematic process employed in this research step by step. Ultimately, we aim to develop an integrated theoretical model.

This study represents the initial application of the grounded theory approach to establish a consumption pattern. By employing this method, we can address questions that quantitative research methods may not effectively answer. These crucial questions pertain to the reasons, conditions, context, and process of using and even quitting these substances. With the grounded theory approach [20], we intend to explore and answer these questions, ultimately presenting a model that explains smokeless tobacco use among adults.

### 2.2 Study participants

The study was conducted in Chabahar city, located in the Sistan and Baluchestan province of southeastern Iran, where high rates of smokeless tobacco consumption among the local population were observed. This region is home to various tribes, with residents predominantly speaking the Baluchi and Jadgali dialects. The majority of individuals in the area practice Islam. To gather data, we visited different locations within the city and its outskirts, carefully selecting suitable venues such as workplaces (e.g., shops) and residential areas (e.g., homes) to ensure the comfort of participants. Each interview began after explaining the research purpose to individuals who used smokeless tobacco, with their voluntary participation and consent. We made efforts to interview individuals of both genders from diverse social and economic backgrounds. However, it is important to note that due to cultural conditions in Chabahar, women who use smokeless tobacco declined to participate in the interviews, which constitutes one of the limitations of our study.

### 2.3 Data collection

The inclusion criteria for study participants consisted of adults aged 18–64 years who were permanent residents of Chabahar. To be eligible, participants had to have a history of using at least one type of smokeless tobacco such as Pan, Pan Parag, Gotkah, BT, Nass, Supari, or other similar products. They also had to be current users, having used smokeless tobacco for at least the past month. Former users of smokeless tobacco were also included and interviewed. Exclusion criteria were based on participants' reluctance to continue with the interview for any reason.

For participant selection, a two-step sampling approach was employed. Initially, purposive sampling was utilized to purposefully select individuals who currently used smokeless tobacco. Subsequently, theoretical sampling, a key aspect of grounded theory, was applied. Theoretical sampling involves strategically selecting new participants, sites, or events to gather data that enriches emerging theories. Unlike random sampling, theoretical sampling aims to maximize diversity in perspectives and experiences to enhance the richness of the data. It serves multiple purposes within grounded theory: supporting theoretical development by gathering data that either supports or challenges emerging concepts, exploring deviant cases, refining emerging theories, and achieving theoretical saturation by ensuring a comprehensive inclusion of data that contributes to the completeness of the emerging theory.

We employed theoretical sampling to support the emergence and saturation of concepts, categories, and subcategories used to construct an integrated model. Ultimately, we conducted interviews with 30 adults aged 18 to 64, and their details are provided in Supplementary No. 1.

### 2.4 Data analysis

According to Strauss and Corbin's perspective, data analysis in this study was conducted in four stages: Analyzing Data for Concepts, Analyzing Data for Context, Bringing Process into the Analysis, and Integrating Categories. Each step will be explained separately. The analysis techniques employed included asking questions, constant comparisons, theoretical comparisons, theoretical sampling, as well as writing memos and creating diagrams [19,21].

An interdisciplinary team of eight researchers participated in this research project. The workload was distributed among team members based on their respective roles. Two researchers played a pivotal role in study design, proposal development, generating initial ideas, and overseeing the overall research process. They also took responsibility for drafting the article, which involved tasks such as coding and establishing a conceptual framework using memos and diagrams.

Additionally, two researchers were involved in conducting interviews and collecting data. They closely interacted with participants, recorded interviews, managed logistics, prepared materials, wrote memos and observations, and captured photographs of the research environment.

Furthermore, two researchers actively engaged in theoretical sampling and the development of the initial theory. They employed a systematic approach based on the grand theory, and their expert opinions greatly contributed to the research process.

Finally, two researchers contributed to writing the project work report and editing the article. They worked under the supervision of the project lead, who held primary responsibility for the project. Throughout the research, all team members actively participated in joint meetings, exchanging ideas, and fostering a scientific and collaborative environment to produce a valuable scientific report.

#### 2.4.1 Analyzing Data for Concepts

All the interviews were recorded, and each interview was carefully listened to multiple times and transcribed. The coding and analysis process began from the initial interviews and continued until the completion of the study.

During the analysis, the data were broken down into smaller units, and coding was applied to identify patterns and themes within the data. We engaged in thoughtful contemplation and conceptualization based on these coded segments. Memos [24] were also written to capture important insights and reflections from certain interviews. Furthermore, we assigned labels to specific data to aid in organizing and categorizing the information. As the number of interviews increased, we utilized diagrams to connect smaller concepts and form larger, overarching concepts.

Additionally, we employed constant comparison between the assigned codes and labels to ensure accurate naming and to facilitate the extraction of meaningful data and concepts. Throughout this process, the labels were revised multiple times to accurately reflect the essence of the data.

#### 2.4.2 Analyzing Data for Context

An analysis of smokeless tobacco consumption conditions was conducted, encompassing both micro and macro perspectives. The micro analysis focused on the daily life conditions of smokeless tobacco users, while the macro analysis examined historical and social contexts. To gain a deeper understanding of the background factors, such as historical, social, and political situations, a thorough investigation was necessary.

Additionally, memos were written to document the existing conditions in the field. These memos aimed to address questions such as why smokeless tobacco consumption occurs, why people engage in it, and what factors influence individuals' consumption patterns. They also explored the processes through which consumption is formed, the locations where it takes place, and the resulting consequences for individuals. This step involved utilizing theoretical sensitivity, which was enhanced by immersing ourselves in the data.

#### 2.4.3 Bringing Process into the analysis

In the subsequent phase, the analysis delved into the process of smokeless tobacco consumption. A process encompasses the continuous flow of actions, emotions, and interactions that occur in response to events, issues, or as part of achieving goals. In this particular research, we sought to identify the actions and reactions of individuals who use smokeless tobacco throughout the consumption process. For instance, we examined how people initiated and sustained tobacco use based on their emotions and beliefs, as well as the role of others and interactions in shaping their consumption patterns. Additionally, we explored the strategies and solutions typically employed by these individuals, such as techniques used to quit or reduce consumption or mitigate the associated side effects.

The first step in this process involved reviewing the notes from the previous analysis. Based on these memos, a summary was written to ensure that the desired process aligns with the identified actions, feelings, and interactions. At this stage, we took a step back to gain a broader perspective and hypothesize. To establish the relationship between certain key categories, the conditional-consequence matrix was employed.

#### 2.4.4 Integrating Categories

In the subsequent step, the categories were merged and composed. Composition involves connecting layers around the central layer, modifying and organizing the resulting theoretical structures. Following the approach outlined by Corbin and Strauss in 2008, the main category represents the basic social process or any other process (educational, legal, spiritual, psychological, etc.). The central category highlights the concept emphasized by the researcher. Both the central concept and other related concepts were derived from the data, enabling the transition from description to conceptualization.

During this phase, we utilized the main categories and subcategories as the foundation for constructing the theory. We revisited and analyzed the various memos we had written on the main and secondary concepts. To refine the details of each category, we examined the interrelationships among the concepts. Continuous interaction between the data and the analyst persisted throughout this stage. Based on this process, we developed a conceptual framework and identified any gaps in logic or clarity. If any gaps were identified, they were re-evaluated, and all memos were utilized to complete and organize the information under each main category.

In this stage, we employed techniques such as re-reading memos, summarizing the main storyline, creating diagrams, and engaging in reflective thinking. Ultimately, a composite diagram was drawn to represent the integrated framework.

### 2.5 Ethical consideration

This study commenced after obtaining ethical approval from the Ethics Committee of Iran University of Medical Sciences. The study was assigned the ethics committee code number IR. IUMS.REC.1398.843. Prior to conducting the interviews, we provided a concise explanation to the participants regarding the research purpose and methodology. We emphasized that their participation in the study was entirely voluntary, and they could withdraw their involvement at any point if they were unwilling to continue (for any reason). The interview location and time were agreed upon with the participants. We assured them of the confidentiality and preservation of their information and recorded files. We also clarified that the study's results would be presented without disclosing their identities. Throughout the study, we addressed any questions the participants had, and the informed consent form was completed by each participant.

#### 2.5.1 Techniques to enhance trustworthiness

Creswell (1998) and Creswell & Miller (2000) proposed eight procedures to achieve what Lincoln and Guba (1985) referred to as “Credibility” and “Trustworthiness” of findings [25]. We have summarized these eight procedures below [26,27].

1. Prolonged engagement and persistent observation in the field: The researcher actively immersed themselves in the findings through continuous engagement and observation, ensuring a deeper understanding of the research context.
2. Negative case analysis: By examining negative cases, the researcher critically analyzed instances that did not align with the emerging patterns, allowing for a comprehensive exploration of the phenomenon.
3. Clarifying researcher bias: To prevent the researcher's personal opinions and biases from influencing the findings, a reflective technique was employed, promoting objectivity in data analysis.
4. Triangulation: The observation method was utilized in data collection, and photographs of the research environment were taken as part of the documentation process. This helped triangulate the data from different sources to enhance its validity.
5. In-member checks: The categories derived from the analysis were shared with some participants, and their feedback was sought to validate the findings, ensuring alignment with their perspectives and experiences.
6. Rich thick description: In order to enrich the research, participants from diverse social levels, educational backgrounds, income brackets, and age groups were invited to participate in the interviews. This contributed to the depth and richness of the data collected.
7. Using peer review or debriefing: Two experts in the field of tobacco reviewed the findings and provided their valuable insights and comments, adding rigor and credibility to the research.
8. External audits: External scrutiny of the research process and findings was conducted to ensure the research met rigorous standards and to enhance the overall trustworthiness of the study.

In this study, the direction and dimensions of the analysis were guided by the research data itself. The researchers employed their own strategies for probing the data. Data collection continued until no new findings emerged, indicating that the saturation point had been reached [26,27].

## 3 Results

The table above (Table 1) presents the main categories, subcategories, and basic concepts derived from the coding process. These categories and concepts were obtained through continuous questioning and comparative analysis of the data collected from the participants. In total, 23 concepts, 6 sub-themes, and 2 themes were identified. The two main categories are “Context of Consumption” and “Need for Effective Supervision”. For further details and explanations regarding the categories and concepts presented in Table 1, please refer to our previously published article [9].

**Table 1:** Main category and the subcategory and codes emerged from the interviews.

category	subcategory	codes
Context of consumption	Consumption Culture Consumption by family members and friends	Traditional rituals Proximity to the culture of the neighboring country Entertainment Consumption after eating Consumption at work The role of religion
	Common and individual beliefs	Belief in the usefulness of consumption Belief about the difficulty of quitting Belief about the ineffectiveness of consumption Belief about the harm of consuming
	Smokeless tobacco use in families and friends	Consumption among Friends and peers Consumption among adults Consumption in childhood Consumption among women
Need for effective supervision	Profitable market	High profits for sellers High demand from the consumer Imposing a cost on the consumer Abundant production in the province and abroad
	Inadequate Supervision	Weak formal and informal social control Pollution for the environment Prohibition of consumption in some formal institutions
	Easy access	Cheap and plentiful Variety of materials available Variety of available consumables

Based on the coding process and the identified concepts and subcategories, three main categories have emerged: “Starting to Use,” “Continued Use,” and “Cessation Attempts” (Table 2).

**Table 2:** The main category and the subcategory of the formation in process of smokeless tobacco consumption.

Categories	Sub Categories	Concepts
Starting to use	Initiation of Smokeless Tobacco Use	The (unpleasant) experience of first use Use to treat toothache To feel big and strong Interaction with the consumer community
Continued use	A sense of euphoria	Feelings of relaxation and euphoria The constant presence of smokeless tobacco in the mouth experience positive moods
	Continuous use of any smokeless tobacco product	Acceptability Social reinforcement Increased consumption Dependence on smokeless tobacco
Cessation attempts	Looking for a new way to quit	Trying to quit Problems and complications of stopping consumption Looking for ways to reduce side effects Obstacles in social relations
	Failure in quit attempts	Failure in quitting Comparisons between different products by users Consumer Preferences with fewer side effects Strong temptation

## 4 Discussion

**Fig. 1:**
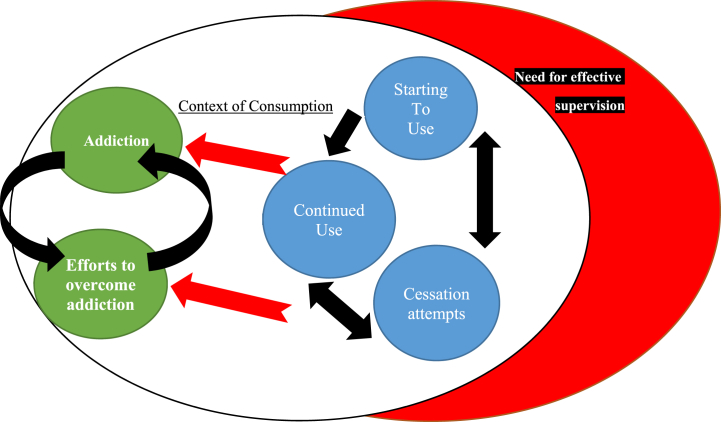
Dependency cycle model in consumption.

The findings of this study have led to the development of a consumption cycle model **(**Fig. 1**)**, significantly enhancing our understanding of the formation of consumption habits. By exploring participants' lived experiences of using smokeless tobacco (SLT) within their specific contexts, the study reveals the trajectory through which individuals enter into the use of smokeless tobacco. These experiences vividly illustrate that people enter the cycle of dependency, sometimes willingly and sometimes unwillingly. This model deepens our comprehension of the process of consumption formation.

Two categories, namely “Inadequate Supervision” and “Context of Consumption,” are identified as factors that potentially contribute to increased use of smokeless tobacco among individuals. In other words, individuals who have the context of consumption and lack supervision within their social environment are more likely to engage in smokeless tobacco use. These categories are outlined in Table 1.

The “Starting to Use” category represents an experience that commonly occurs among users. First-time usage experiences, using smokeless tobacco to alleviate toothache, and the desire to feel more mature and powerful, as well as increased interaction with the consumer community, are mentioned by participants. Many individuals reported that their initial experience was unpleasant, but subsequent usage led to addiction and habit formation over time.

The “Continued Use” category includes two subcategories: a sense of euphoria and ongoing use of smokeless tobacco products. The “Cessation Attempts” category comprises two subcategories: seeking new methods to quit and experiencing failure in quit attempts. The consequences of smokeless tobacco consumption are depicted in the “Addiction” and “Recovery from Addiction” categories.

Regarding the extracted subcategories, customs and rituals are identified as background factors in this study. These factors have a significant influence on other aspects. Many of these customs have been passed down through generations, with grandparents narrating stories about the consumption of these substances to younger individuals and teenagers. Additionally, spittoons or containers for collecting saliva are used in social gatherings.

The influence of rituals and customs on tobacco use is also highlighted in a study conducted by Shikar Grover et al. in India, which reports the social acceptability and widespread use of smokeless tobacco in rural areas [28]. Furthermore, two other studies conducted in different countries support the concept of customs and rituals identified in our study [29,30].

The process categories identified in this research include a sub-category known as the “unpleasant experience of first consumption,” which was a significant concept expressed by the majority of consumers. It involved a range of symptoms such as severe headaches, nausea, vomiting, deep sleep, fainting, and, in some cases, the need for emergency medical care and hospitalization. Despite experiencing these symptoms during the initial use, most users still chose to continue using smokeless tobacco due to various reasons. One of the reasons mentioned was the belief that such side effects would only occur during the first use and not in subsequent uses. This finding is unique to our study and has not been reported in other articles. The possible explanation for this discrepancy could lie in the chemical composition of smokeless tobacco products in Iran.

The second main category within the process categories is “Continued Use.” Users typically engage in repeated consumption sessions, which can last anywhere from 5 to 10 min up to 2 h. A study conducted by P. Soto et al. supports our findings, stating that smokeless tobacco consumption leads to increased activity, a sense of euphoria, heightened alertness and attention, and feelings of relaxation during chewing [31]. The concepts associated with the process of smokeless tobacco consumption include feelings of relaxation and euphoria, continuous presence of smokeless tobacco in the mouth, experiencing positive moods, social acceptability and reinforcement, increased consumption, and dependence on these substances (Table 2).

The constant presence of smokeless tobacco in the mouth is a practice observed among some individuals during the interviews. Some participants admitted that they enjoy this habit. However, others mentioned that they engage in this behavior primarily due to their addiction, as they feel the need to constantly have it in their mouths to sustain their pleasure. It is worth noting that in a study conducted by Arvind Motokrishnan et al. this behavior was reported as an unhygienic and harmful habit for oral health, but many consumers in our research seemed to overlook this health concern [13].

The concept of experiencing positive moods, as expressed by the majority of participants, highlights important points. Individuals with less severe addiction often mentioned feeling refreshed, while those with more severe addiction reported feelings of tiredness and difficulty performing certain tasks due to excessive consumption. People's sense of cheerfulness tends to be higher in the early years of consumption, but as they age and experience decreased physical strength and complications associated with consumption, they no longer perceive the use of these substances as cheerful. This finding aligns with Scott D's study, which reported an inverse relationship between tobacco use and physical activity levels, supporting our findings [32].

The concept of acceptability, as identified in our study, highlights the fact that individuals who consume smokeless tobacco more frequently often started their consumption during adolescence. They stated that using these substances helped them gain better acceptance within their peer community. This finding is consistent with a study conducted by Kumar Sah et al. where 22 % of individuals considered being sociable as a factor and reason for using smokeless tobacco [33]. Another study by Irfan et al. also mentions the role of friends and peers in encouraging consumption, which aligns with our findings [34].

The concept of dependence on smokeless tobacco is an important finding from our study. It leads to continuous consumption, as individuals feel a sense of improvement and psychological preference to maintain that state. Some participants mentioned that they continued using smokeless tobacco to avoid discomfort and forget the difficulties of life. At times, they stated that they used it as a means to relieve fear and stress. In some cases, individuals were so dependent on smokeless tobacco that they would consume it before engaging in conversations to feel more at ease. A study conducted by Chellappa et al. reported a nicotine dependence rate of 53.57 % among users of smokeless tobacco [35] while other studies have also reported similar levels of dependence [36,37]. These findings align with the concept of dependence extracted from our research.

The category of “Cessation attempts” comprises two subcategories and eight concepts, each of which will be discussed separately. The first concept is “Trying to quit,” which was expressed by the majority of participants with years of experience in using smokeless tobacco. This concept reflected the exhaustion and disgust felt by individuals who were addicted. Many of them actively sought ways to quit and had made various attempts in the past. They explored traditional methods of quitting as well as consulting doctors and even trying medications that had become available for quitting. Some individuals were specifically looking for a method or medication that would completely eliminate their temptation to consume more. A study by Ahluwalia et al. which involved 31 countries and focused on quitting behaviors and the use of cessation services, revealed that an average of 74.4 % of people attempted to quit without any external assistance, with only around 2 % seeking help via phone consultations [38]. Similarly, our study found that a significant percentage of the participants attempted to quit without seeking assistance from others [38].

The concept of “problems and complications of stopping consumption” was expressed by individuals who were attempting to quit. They reported facing difficulties in performing their daily tasks without consuming smokeless tobacco. Severe addiction to these substances resulted in symptoms such as headaches, drowsiness, restlessness, and irritability. They described the experience as feeling like something was missing.

The concept of “looking for ways to reduce side effects” emerged from the participants' narratives. Some individuals mentioned reducing their consumption as a strategy, while others sought alternative forms of smokeless tobacco. For instance, one person mentioned sucking on the substance instead of chewing it to make it last longer in their mouth, while another preferred one type of smokeless tobacco over another. Some participants believed that avoiding swallowing saliva after consumption could prevent stomach damage and kidney stones. Additionally, many found it beneficial to brush their teeth after consuming smokeless tobacco to mitigate oral damage. Some individuals even mentioned using a Baluchi toothbrush for this purpose. These solutions were commonly expressed by participants during the interviews.

Another study reported an increase in the prevalence of smokeless tobacco use over a ten-year period, particularly in Southeast Asia, where smokeless tobacco use is becoming an epidemic and replacing cigarette use. One of the reasons for this shift may be the perception that smokeless tobacco is less harmful [39].

The concept of “obstacles in social relations” emerged as a significant factor influencing individuals' desire to quit smokeless tobacco. One obstacle mentioned was the difficulty of attending events and gatherings where other people were not consumers. In such situations, it was challenging for them to use smokeless tobacco since they would need to expel saliva after consumption. On the contrary, casual gatherings with other consumers often provided a container for collecting saliva.

Another obstacle identified was the issue of having a full mouth while consuming. This often resulted in unclear speech and difficulty in communicating effectively. Additionally, the problem of bad breath and the unsightly appearance of teeth, characterized by black and red spots caused by excessive consumption, posed challenges for individuals in interacting with others. These factors were perceived as obstacles that motivated people to seek ways to quit.

The concept of “failure in quitting” was expressed by the majority of individuals who had attempted to quit smokeless tobacco multiple times. Some reported attempting to quit for a week, a month, two months, and in rare cases, even a year or two before relapsing. According to their accounts, the main reason for these failures was their personal level of desire, as they were unable to completely quit. This finding aligns with the study conducted by Irfan et al. which reported that the majority of users continue to use smokeless tobacco in a continuous cycle of attempts to quit, relapse, and recovery. These findings are consistent with the concepts extracted in our research [34].

The concept of “comparisons between different products” emerged from the interviews with participants. Each individual had their own criteria for comparison. Some based their comparison on price, while others focused on taste, endurance, or even compared it to other products such as hookah and cigarettes. These comparisons served as a way for individuals to justify and convince themselves that their chosen method of consumption was suitable.

The concept of “consumer preferences with fewer side effects” is an important factor contributing to the failure to quit smokeless tobacco. Individuals who hold this belief are less motivated to quit and tend to underestimate the significance of the side effects associated with consumption. They often believe that these side effects do not apply to them or that they will not experience any long-term problems. For instance, some individuals may prefer certain types of smokeless tobacco, such as nas or supari, believing that they have fewer side effects compared to other types like panparag or gutkah. These preferences are influenced by personal experiences, observations of side effects in themselves or others, and societal comparisons that deem these substances as less dangerous than other narcotics. Such comparisons and preferences contribute to a lack of motivation to quit and can even lead individuals to relapse if they attempt to quit. A study by Ahluwalia et al. on quitting behaviors showed that some adults opt to replace smoked tobacco with smokeless tobacco as an individual strategy to quit smoking. This finding indicates that the preference for substances with fewer side effects is not limited to smokeless tobacco alone, but also applies to smoked tobacco, underscoring the consistency of our study results [40].

“Strong temptation” emerged as a prominent concept during the interviews with participants, and it was identified as a significant factor contributing to the failure to quit smokeless tobacco. According to the majority of participants, this temptation is so intense that it consumes their thoughts, making them solely focused on obtaining and consuming these substances again. Some participants even mentioned that individuals who cannot afford to purchase the substances may resort to theft in order to obtain the money needed. This observation aligns with the findings of a study conducted by Tas et al. which examined the temptation scale and highlighted the influential factors that drive individuals to revert back to their old habits when faced with challenging situations. The study provides evidence of the presence of temptation among individuals who use smokeless tobacco and attempt to quit, which ultimately leads to unsuccessful quit attempts [41]. For the analysis of consequences, two main categories were identified: addiction and efforts to overcome addiction. The concepts presented in Table 3 will be discussed individually.

**Table 3:** Main categories and subcategories of the consequences of smokeless tobacco consumption.

Categories	Sub Categories	Concepts
Addiction	Severe addiction	Permanent addiction Psychological dependence
	Side effects	Serious physical complications Injuries to teeth, gums and mouth
Efforts to overcome addiction	Quit	Permanent Cessation Short term cessation Cessation with an alternative method of Consumption
	Death	Premature deaths Slow death

Permanent addiction primarily refers to the strong physical and biological craving to consume these substances on a daily basis. Physical dependence may not become evident until the use is continued. However, if the substance is not consumed, addiction symptoms become apparent. A similar finding of severe addiction to smokeless tobacco consumption was reported in a study by Sotto et al. which aligns with the results of our research. Participants explicitly expressed their dependency on smokeless tobacco use [31].

For the concept of Psychological dependence, many participants implicitly mentioned their psychological experiences. They perceived consumption as a source of relaxation, satisfaction, happiness, and enjoyment. Some even described it as a means to feel stronger and to perform tasks correctly. Several consumers stated that when they do not use it, they become aggressive, bored, and anxious. In a study conducted by Ahsan et al. out of 310 smokeless tobacco users, 63 % exhibited high dependence, 29.4 % had moderate dependence, and 6.85 % had low dependence on smokeless tobacco. These findings support the results of our study [42].

The concept of Serious physical complications includes various conditions such as cancer, kidney stones, cardiovascular diseases, diabetes, and complications related to childbirth. Furthermore, the concept of Injuries to teeth, gums, and mouth encompasses damages such as infections, tooth decay, mouth and jaw injuries, and tooth loss. These consequences resulting from consumption have been extensively discussed in numerous studies [[43], [44], [45], [46]].

The concept of Permanent Cessation emerged from the interviews, indicating that some individuals were able to quit using smokeless tobacco and had no desire to resume consumption. These individuals expressed satisfaction with their new condition and reported feeling healthy. We refer to this concept as “Permanent Cessation,” as these individuals had successfully abstained from tobacco use for many years. It is noteworthy that most of these individuals achieved cessation through self-treatment at home, as access to standard quit programs was limited, particularly for low-income participants. A similar study conducted in India also reported a lack of access to standard treatment programs among low-income individuals [47].

Another concept that emerged from numerous interviews was the concept of Short-term Cessation. This refers to individuals who were able to quit using smokeless tobacco for a short period, ranging from 2 weeks to a month or even up to 2 years. However, for various reasons, these individuals eventually resumed their tobacco use. Many participants reported experiencing withdrawal symptoms during their period of abstinence but eventually relapsed. A study conducted by Venkatesan et al. highlighted the existing gaps in understanding smokeless tobacco addiction, dependence, and intervention programs. This gap in support and intervention was also evident in our study, as individuals who had successfully quit for a short period ultimately returned to using tobacco due to the lack of adequate support. This finding is consistent with the results of our study [47].

As indicated in Table 3, the concept of “Death” is a subcategory that emerged from the interviews. This concept refers to cases where individuals experienced premature death, often attributed to high consumption of smokeless tobacco or consuming it during intense physical activities. Such cases have been associated with various health complications, including strokes, heart diseases, kidney problems, and lung issues. The category was named “Death” because it represents instances where individuals, particularly young people, passed away earlier than expected due to their tobacco use.

Supporting this concept, a study conducted by D. N. Sinha et al., in 2010 reported a significant number of deaths related to smokeless tobacco use in India. The study estimated that approximately 368,127 deaths (217,076 women and 151,051 men) were attributable to smokeless tobacco use, with nearly 60 % of these deaths occurring among women. This research highlights the alarming impact of smokeless tobacco on mortality rates, particularly in terms of premature death, and the statistical evidence further validates the significance of the concept of premature death identified in our study [48].

The final concept within the subcategory of “Death” is “Slow death.” This concept pertains to the long-term consequences of using smokeless tobacco, leading to the development of chronic diseases such as diabetes, high blood pressure, and various types of cancers. The continuous use of smokeless tobacco can progressively damage vital organs such as the heart, kidneys, and lungs. Individuals who have been using smokeless tobacco for an extended period of time often become aware of these damages when they experience signs and symptoms associated with these health issues. Aging, severe addiction, and the resulting disabilities, coupled with the lack of effective treatment options, contribute to a slow deterioration of health, which can ultimately be considered a form of “slow death."

This concept has also been addressed in previous studies, highlighting the long-term health risks and the overall negative impact of smokeless tobacco use on individuals' well-being and quality of life [49,50].

### 4.1 Strengths and limitations

This study contributes to the field by qualitatively developing a model that explains smokeless tobacco consumption. The model can provide valuable insights for policymakers, psychologists, social workers, and health activists seeking to reduce the consumption of smokeless tobacco. A strength of this study is the researchers' extensive experience in this field, which is crucial for qualitative research.

However, there are several limitations to consider. One limitation is the gender imbalance in the participant pool, as only male participants were included in the interviews. This was due to cultural factors and constraints that prevented the inclusion of female participants. Despite efforts to involve women by having a female interviewer, social stigma, lack of permission from husbands, fathers, or brothers hindered their participation.

Another limitation was the unwillingness of some participants to have their interviews recorded, leading to their exclusion from the study. Concerns about potential consequences, such as being stigmatized as an addict or patient, influenced their decision to decline participation. Additionally, finding a suitable and comfortable location for interviews posed challenges, as some participants preferred not to be interviewed at their workplace.

The non-cooperation of certain participants was another limitation, resulting in their exclusion from the study. Their insights and information could have added further depth to the understanding of the topic.

For future studies, it is recommended to employ strategies and measures that promote the participation of female participants in research interviews, considering the limitations mentioned above.

It is worth noting that previous studies on smokeless tobacco predominantly focused on the risks and impacts for men, with some studies examining both sexes collectively. Smokeless tobacco use tends to be more prevalent among male adults and youth compared to women [46].

## 5 Conclusion

The extracted pattern is derived from data and the lived experiences of the participants, providing insights into the process of smokeless tobacco consumption. This pattern elucidates the factors influencing its formation and the resulting consequences. By identifying these aspects, it enables the recognition of existing issues and the formulation of solutions. The Dependency Cycle Model in consumption can serve as an effective framework for researchers to guide their interventions related to smokeless tobacco use. This model facilitates a comprehensive understanding of the complex dynamics involved and aids in devising strategies to mitigate the negative impacts associated with smokeless tobacco consumption.

## Ethics statement

This study commenced after obtaining ethical approval from the Ethics Committee of 10.13039/100012021Iran University of Medical Sciences. The study was assigned the ethics committee code number IR. IUMS.REC.1398.843.

## Data availability statement

Data included in article/supp. Material/referenced in article.

## Additional information

No additional information is available for this paper.

## CRediT authorship contribution statement

**Esmaeil Fattahi:** Conceptualization, Data curation, Formal analysis, Funding acquisition, Investigation, Methodology, Project administration, Resources, Software, Validation, Visualization, Writing – original draft, Writing – review & editing. **Mahnaz Solhi:** Conceptualization, Data curation, Formal analysis, Funding acquisition, Investigation, Methodology, Project administration, Resources, Software, Supervision, Validation, Visualization, Writing – original draft, Writing – review & editing. **Seyed Saeed Hashemi Nazari:** Conceptualization, Data curation, Formal analysis, Investigation, Methodology, Project administration, Resources, Software, Supervision, Validation, Visualization, Writing – original draft. **Hadis Barati:** Conceptualization, Data curation, Formal analysis, Funding acquisition, Investigation, Methodology, Resources, Software, Validation, Visualization, Writing – original draft, Writing – review & editing. **Fardin Mehrabian:** Conceptualization, Data curation, Formal analysis, Funding acquisition, Investigation, Methodology, Resources, Validation, Visualization, Writing – original draft. **Zahra Sadat Manzari:** Conceptualization, Data curation, Formal analysis, Funding acquisition, Investigation, Methodology, Project administration, Resources, Software, Supervision, Validation, Visualization, Writing – original draft. **Iraj Zareban:** Conceptualization, Data curation, Formal analysis, Funding acquisition, Investigation, Methodology, Resources, Software, Validation, Visualization, Writing – original draft. **Abolhasan Afkar:** Conceptualization, Data curation, Formal analysis, Funding acquisition, Investigation, Methodology, Resources, Software, Validation, Visualization, Writing – original draft, Writing – review & editing.

## Declaration of competing interest

The authors declare that they have no known competing financial interests or personal relationships that could have appeared to influence the work reported in this paper.
