# Supplementary material for: A model to explain smokeless tobacco consumption in adults: A grounded theory study
Source: Heliyon. 2023 Oct 13;9(10):e20734. doi: 10.1016/j.heliyon.2023.e20734 (PMC10589854; doi:10.1016/j.heliyon.2023.e20734)
Supplement: Multimedia component 1 [file mmc1.docx]

| Supplementary No. 1.**Demographic information of samples** | | | | | | | | | | | |
| --- | --- | --- | --- | --- | --- | --- | --- | --- | --- | --- | --- |
| **Duration of use** | **Type of consumption** | **Frequency of consumption per day** | **interview duration** | **interview location** | **number of children** | **Marriage** | **Occupation** | **Level of education** | **age** | **Gender** | **Participant**  **N** |
| 10 | Pan Parag | 5 | 25 | city center area | - | Single | Carpenter | Senior high school | 27 | man | 1 |
| 13 | pan | 2 | 25 | city center area | 4 | Married | worker | Senior high school | 30 | man | 2 |
| 10 | Pan  gutkha | 20 | 26 | city center area | 2 | Married | Three Wheel Motorcycle driver | Junior high school | 30 | man | 3 |
| 9 | gutkha | 6 | 20 | city center area | - | Single | worker | Junior high school | 26 | man | 4 |
| 5 | Naswar | 7 | 15 | suburb | - | Single | worker | Junior high school | 20 | man | 5 |
| 43 | Naswar | Always | 30 | village | 4 | Married | Farmer | illiterate | 55 | man | 6 |
| 13 | pan supari | 4 | 20 | city center area | 3 | Married | Carpenter | illiterate | 32 | man | 7 |
| 1 | gutkha | 5 | 22 | city center area | - | Married | worker | illiterate | 33 | man | 8 |
| 15 | Pan Parag | 3 | 21 | city center area | 2 | Married | worker | Junior high school | 25 | man | 9 |
| 4 | Gutkha supari | 4 | 25 | city center area | - | Single | worker | Junior high school | 18 | man | 10 |
| 15 | Naswar | 3 | 20 | suburb | 2 | Married | Shopkeeper | Elementary | 31 | man | 11 |
| 13 | Naswar | 4 | 25 | suburb | 3 | Married | smokeless tobacco seller | illiterate | 33 | man | 12 |
| 10 | Naswar | 6 | 25 | suburb | - | Single | Fruit seller | Junior high school | 21 | man | 13 |
| 5 | Pan Parag | 15 | 15 | suburb | - | Single | Unemployed | Junior high school | 24 | man | 14 |
| 10 | mawa | Always | 15 | city center area | 5 | Married | Shopkeeper | Junior high school | 40 | man | 15 |
| 7 | Pan Parag  gutkha | 7 | 17 | village | 1 | Married | repairman | Junior high school | 27 | man | 16 |
| 5 | gutkha | 5 | 15 | city center area | 2 | Married | Building Painter | Elementary | 30 | man | 17 |
| 5 | supari | 2 | 18 | city center area | 4 | Married | Employee | High education (university) | 50 | man | 18 |
| 25 | gutkha | 8 | 18 | city center area | 3 | Married | Employee | High education (university) | 34 | man | 19 |
| 15 | BITI  Pan parag | 6 | 19 | suburb | - | Divorced | Building Painter | Elementary | 37 | man | 20 |
| 12 | gutkha | Always | 24 | city center area | 3 | Married | Three Wheel Motorcycle driver | Elementary | 33 | man | 21 |
| 25 | gutkha | Always | 18 | city center area | 3 | Married | Employee | High education (university) | 34 | man | 22 |
| 3 | gutkha | 8 | 16 | suburb | 2 | Married | masonry works | illiterate | 58 | man | 23 |
| 2 | Pan Parag | Always | 15 | suburb | - | Single | Unemployed | Senior high school | 19 | man | 24 |
| 10 | supari | Always | 20 | suburb | - | Single | Fisherman | Senior high school | 18 | man | 25 |
| 2 | pan | Always | 24 | suburb | - | Single | Fisherman | Senior high school | 19 | man | 26 |
| 4 | supari | Always | 25 | suburb | - | Single | Fisherman | Senior high school | 18 | man | 27 |
| 12 | gutkha | 6 | 20 | suburb | 2 | Married | Three Wheel Motorcycle driver | Senior high school | 28 | man | 28 |
| 10 | Mawa | Always | 18 | city center area | 2 | Married | repairman | Junior high school | 30 | man | 29 |
| 13 | Pan Parag, | 10 | 15 | city center area | - | Single | Building Painter | Junior high school | 29 | man | 30 |
